# Long-acting injectable antipsychotics: Six-month follow-up of new outpatient treatments in Bologna Community Mental Health Centres

**DOI:** 10.1371/journal.pone.0211938

**Published:** 2019-02-15

**Authors:** Lorenzo Berardi, Ippazio Cosimo Antonazzo, Carlo Piccinni, Emanuel Raschi, Emanuele Forcesi, Angelo Fioritti, Domenico Berardi, Fabrizio De Ponti, Antonella Piazza, Elisabetta Poluzzi

**Affiliations:** 1 Department of Medical and Surgical Sciences, University of Bologna, Bologna, Italy; 2 Department of Biomedical and Specialty Surgical Sciences—Clinical psychiatric division, University of Ferrara, Ferrara, Italy; 3 Department of Mental Health, Local Health Authority of Bologna, Bologna, Italy; 4 Department of Biomedical and Neuromotor Sciences, University of Bologna, Bologna, Italy; Wayne State University, UNITED STATES

## Abstract

**Purpose:**

This study aims to describe factors associated to treatment continuity and psychiatric relapses in patients treated with Long Acting Injectable antipsychotics (LAIs) in Bologna Community Mental Health Centers (CMHCs).

**Methods:**

New LAI treatments administered between July 1, 2010 and June 30, 2015 in CMHCs were selected. The cohort was followed-up for 6 months; predictors of continuity and psychiatric admissions were investigated by using logistic regression- and Cox- analysis respectively.

**Results:**

Among the cohort of 1 070 patients, only 222 (21%) continued LAI treatment during the follow-up. LAI continuity was higher with first generation agents (OR: 1.71, 95%CI 1.18–2.49) and in case of previous psychiatric hospitalizations (OR 2.00, 95%CI 1.47–2.74). Incidence of psychiatric hospital admissions showed a sharp reduction in the follow-up compared with 6-month period before initiation (from 458 to 212), and was associated with previous psychiatric hospitalizations (HR 3.20, 95%CI 2.22–4.59), immigration (HR 3.13, 95%CI 1.28–7.69) and LAI discontinuation (HR 1.14, 95%Cl 1.01–1.97).

**Conclusions:**

Psychiatric hospital admission before LAI initiation was the main predictor both of LAI continuity and hospitalization during the follow-up.

## Introduction

The long-acting injectable antipsychotics (LAIs) have aroused new interest, especially with the introduction of second generation agents (SGA) in LAI formulations. Some aspects are still controversial, including delayed recovery from adverse reactions and possible perception of stigma and coercion, which could compromise the quality of the therapeutic relationship [1].

NICE guidelines recommend that treatment with LAIs should be considered, after oral antipsychotic (AP) medication, for patients with psychosis or schizophrenia who would prefer such treatment after an acute episode or when avoiding covert non-adherence to antipsychotic medication is a clinical priority [2]. SGA-LAIs are claimed to have a number of advantages over first-generation long-acting antipsychotics (FGA-LAIs), such as more rapid onset of action [3], prolonged intervals between administrations, better tolerance and lower risk of drug interactions [4, 5]. In everyday clinical practice, there is a rising tendency to use LAIs also for people with non-schizophrenic conditions, such as bipolar [6], personality and behavioral disorders [7]. In fact, a variety of rather expensive SGA-LAIs are now marketed and such a large availability is likely to increase substantially LAIs use [8], notwithstanding the well-known geographical variations in prescription rates [9, 10].

The present study aims to provide real-world evidence on LAI treatments in Community Mental Health Centers (CMHCs) of a Northern Italy area from 2010 to 2015. It is first intended to outline the five-year trend in LAI initiation and to point out if the recent expected LAIs increase in psychiatrists’ prescriptions was observed. Moreover, this study will describe social and clinical characteristics of LAI new users, and will investigate over a 6-month follow-up LAI continuity along with its principal predictors, as well as the relationship of LAI discontinuation and other risk factors with hospitalization.

## Materials and methods

### Setting and data sources

The study was carried out in the Community Mental Health Centers (CMHCs) of the Bologna Local Health Authority (LHA) in Emilia-Romagna Region. Bologna LHA covers both urban (i.e.; the main regional city) and rural areas, and serves approximately 860,000 inhabitants, that represent a fifth of the regional population. CMHCs are run within the Department of Mental Health by multi-professional teams and are responsible for community and residential treatments of adults, as well as for coordinating with hospital psychiatric units and for continuity of psychiatric care.

Data on patients and treatments are recorded in the local mental health information system, established in 2007 for administrative and clinical-epidemiological purposes. Data selected for the present analysis include socio-demographic characteristics, diagnoses, length of CMHC care, hospital admissions and AP medication delivered by CMHC staff. Since in Italy public and private licensed hospitals record admissions and discharges in the Hospital Discharge Register (HDR), the local information system has been cross-checked with the regional HDR, so as to include all admissions for psychiatric reasons, both in the public and the private sector. Oral APs are usually supplied by CMHC, whereas LAI injections are directly administered by CMHC nurses.

Diagnoses are codified according to the International Classification of Diseases, 9th Revision, Clinical Modification (ICD-9-CM) and drugs according to the Anatomical Therapeutic Chemical classification (ATC).

FGA-LAIs marketed in Italy from 2010 to 2015 were fluphenazine decanoate (ATC: N05AB02), haloperidol decanoate (N05AD01), perphenazine enantate (withdrawn in 2011, N05AB03) and zuclopenthixol decanoate (N05AF05). SGA LAIs included risperidone long-acting (N05AX08), paliperidone palmitate (N05AX13), olanzapine pamoate (N05AH03), aripiprazole long-acting (N05AX12). It should be mentioned that only drugs in the Regional Formulary Drugs (RFD) are usually provided by CMHCs, while other marketed drugs can be exceptionally delivered if specific clinical reasons are detailed. Paliperidone entered in the RFD in 2014, while olanzapine and aripiprazole were not yet included in the RFD when the study ended.

The study protocol was approved by the Ethics Committee of Bologna-Imola Local Health Authorities in September 2015. Data extracted from the local mental health information system have been anonymized by the Department of Mental Health office before the analysis; ethics committee waived the requirement for informed consent.

### Study population and design

The study cohort was represented by patients aged 18 years or more, residents in the area of Bologna LHA, receiving a new treatment with LAIs between July 1, 2010 and June 30, 2015 and in contact with CMHC for at least 6 months before starting LAIs. A “new-start LAI treatment” was defined when no LAI had been administered by a CMHC within the previous 6 months. In case of subsequent cycles of LAIs for the same patient over the study period, only the first one was considered. The first LAI administration date was used as a reference to collect socio-demographic and clinical data.

Information on sex, age, nationality, living area (urban vs rural), years of education, employment, marital status and living arrangement (with family, alone, residential setting, other arrangement) were extracted from the local mental health information system. From the same source, we also collected psychiatric diagnoses, grouped into the following categories: schizophrenic-like disorders (ICD9 CM codes: 291.3, 291.5, 292.1, 292.81, 293.81, 293.82, 295, 297–299), bipolar disorders (296.0, 296.1, 296.4–8, 296.90, 301.13), depressive disorders (296.2–3, 296.82, 300.4, 309.0, 309.28, 311), anxiety disorders (300.0–3, 300.7–8, 309.24, 309.81), personality disorders (301, 303.9, 304, 310.1), other diagnoses (other codes not previously listed).

Data on hospital admissions for psychiatric reasons and oral AP utilization (ATC: N05A –antipsychotics, except for N05AN01 –lithium) were collected over the six-month period before the entry date and during the 6-month follow-up together with possible discharge from CMHCs.

Rate of LAI utilization in CMHCs was estimated and each treatment was assessed for continuity along the six-month follow-up: it was considered “discontinued” when either intervals > 45 days between two LAI administrations or switches to other LAIs occurred; all other treatments were considered “continuous”. Discontinued treatments were further distinguished into “definitively discontinued”, if no more LAI administrations were found before the end of the six-month follow-up, and”intermittently discontinued”.

Psychiatric hospital admissions were assumed as a proxy of severe clinical relapses. Data on drug treatments during hospital stay were not available.

### Statistical analysis

Rates of LAI utilization within Bologna CMHCs from 2011–2015 were evaluated.

A descriptive analysis of socio-demographic and clinical characteristics of the study cohort was performed, including psychiatric admissions and oral APs prescribed in the six month-period before initiation. Continuity of LAI medication and hospital admissions during the six-month follow-up were then analyzed.

In order to identify predictors of LAI continuity, a logistic regression model was used including the following characteristics as categorical variables: sex, age, nationality, living status, diagnosis, LAIs generation and previous hospital admission. Any independent variable with an adjusted odds ratio (ORadj) statistically significant (*p*< 0.05) at 95% confidence intervals (95%CI) was considered a predictive factor of treatment continuity.

Cox proportional hazard model was used to calculate hazard ratios and the relevant 95%Cl, in order to assess the impact of LAI discontinuation and other risk factors on hospital admissions. Each individual contributed with person-time to exposure from start of LAI until the first psychiatric hospital admission or to the end of follow-up, whichever occurred first. LAI exposure was analyzed as time-dependent variable: for each patient, each follow-up day was categorized into “on-treatment” vs. “treatment-free” status. Therefore, patients with continuous treatment contributed with the whole time-period to the on-treatment status, whereas discontinued patients contributed to both categories, with different time-windows, on the basis of the length of discontinuation. A multivariate model was performed including the following categorical variables: age, gender, previous psychiatric hospitalization, LAI generation, diagnosis, living arrangement and nationality. Because of the possible latency in LAI onset of action, this analysis was performed either by considering all days of exposure and all hospitalizations, or by excluding a time-window of 15, 30 or 45 days after the new-start LAI treatment both for exposure and hospitalisation.

Statistical analyses were carried out using the Statistical Package for Social Science (SPSS) version 21.0 for Windows.

## Results

### Five-year trends in new LAI treatments

The annual frequency of new treatments with LAIs did not increase from 2011 to 2015, involving on average 2.1% of CMHC patients and 5.4% of patients on AP treatment (Table 1).

**Table 1:** Year-by-year new LAI treatments in Bologna CMHCs.

Year	CMHC patients	CMHC patients with APs	CMHC patients with LAI treatment	New LAI treatments^a^	New LAI treatments in CMHC pts (%).	New LAI treatments in AP pts (%)
**2011**	16,875	6,472	1544	390	*2*.*3*	*6*.*0*
**2012**	17,346	6,877	1580	385	*2*.*2*	*5*.*6*
**2013**	17,135	7,039	1579	353	*2*.*1*	*5*.*0*
**2014**	17,811	6,932	1569	341	*1*.*9*	*4*.*9*
**2015**	17,154	6,958	1610	368	*2*.*2*	*5*.*3*

^a^ in the second semester 2010, 1373 LAI treatment and 200 new LAI treatments were found.

**Fig 1:**
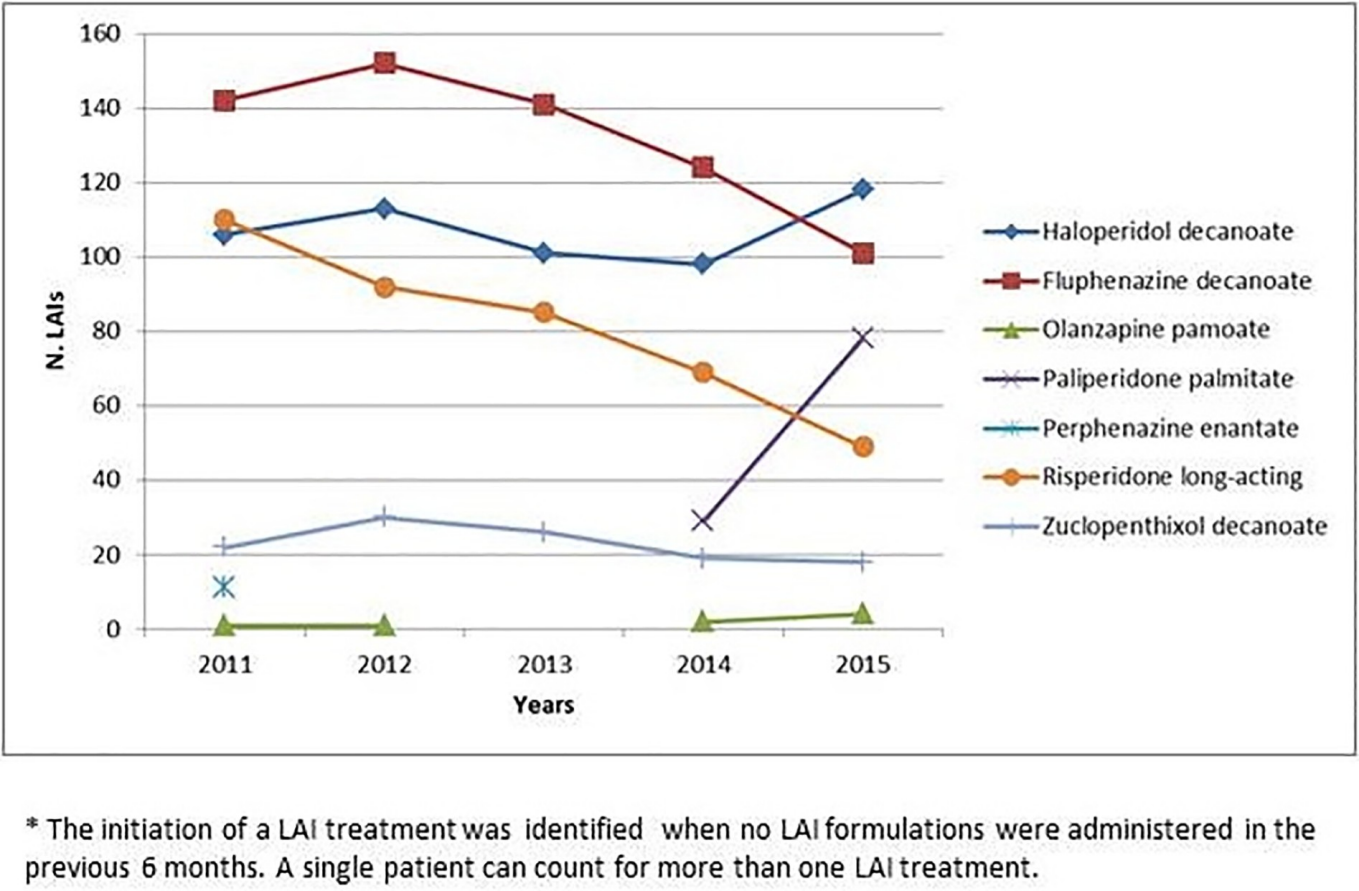
Year-by year initiation of LAI treatments: Trends in prescriptions 2011–2015.

Fig 1 shows the trend in starting LAI treatments. A progressive decrease in fluphenazine (2011: N = 142; 2015: N = 101) and risperidone (2011: N = 110; 2015: N = 49), as well as an increase in paliperidone (no prescriptions until 2013; 2014: N = 29; 2015: N = 78) were observed. Haloperidol showed a fluctuating trend (2012: N = 113; 2014: N = 98 and 2015: N = 118), zuclopenthixol prescriptions remained low and unchanged after a mild increase in 2012 and olanzapine was prescribed only in a few cases, in part because of the need of monitoring patients in adequate health settings due to the risk of post-injection syndrome.

A moderate prescribing shift to SGA-LAIs was found over the five-year period, though FGA-LAIs remained the most frequent option for initiating new treatments, from 71.7% (n = 281 out of 392) in 2011 to 64.4% (n = 237 out of 368) in 2015. In particular, in 2011 the most prescribed formulations were fluphenazine (36%) and haloperidol (27%), whereas in 2015 fluphenazine decreased to 27%, haloperidol increased to 32% and paliperidone attained 21%.

### Socio-demographic and clinical characteristics of the study cohort

**Fig 2:**
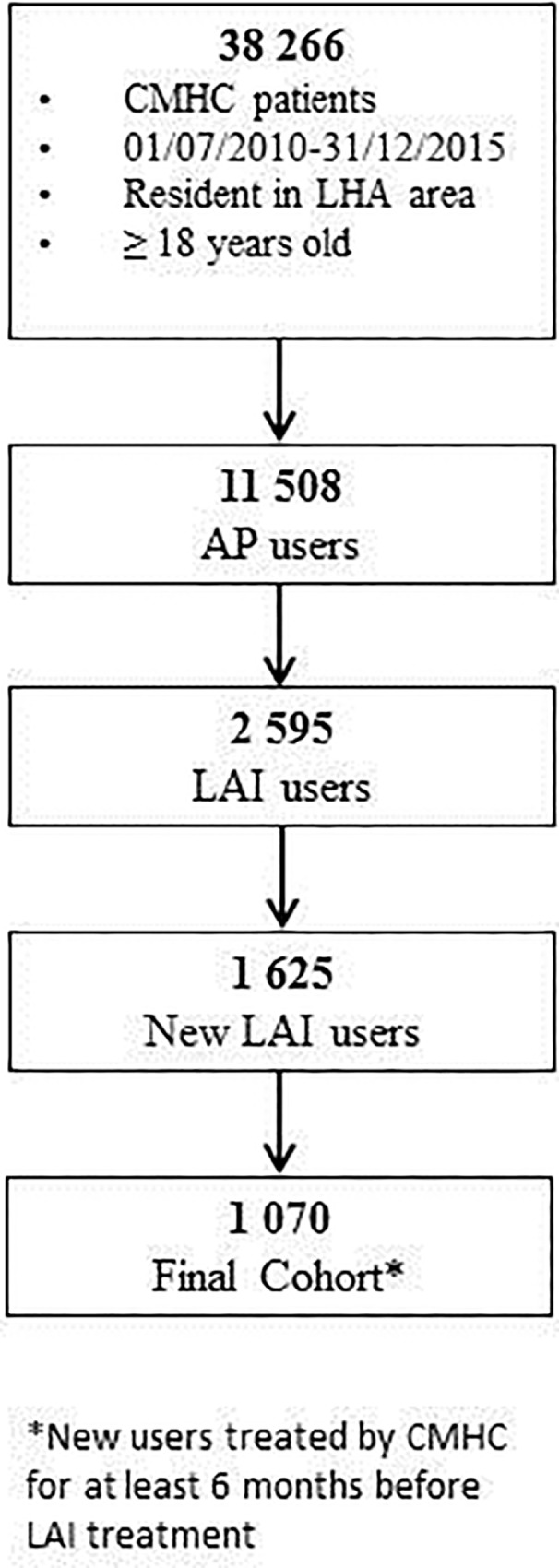
Flow chart of cohort selection.

Among residents aged ≥18 years treated by Bologna CMHCs from 1^st^ July 2010 to 30^th^ June 2015 (38,266 patients), AP users were first selected (11,508 patients) and LAI users were then identified (2,595 patients, 22.5% of AP users). The study cohort included only those subjects who initiated a LAI treatment over the period and had received CMHC care for at least 6 months before then (1,070 patients, 41.2% of LAI users). For patients initiating more than one LAI treatment during the study period (new LAI treatments were 2,037 on 1,625 patients), only the first one was considered (Fig 2).

The characteristics of the study cohort are described in Table 2: males were slightly more frequent than females (52.4% vs. 47.6%), 68% were aged 35–64 years and 92.2% were Italians. The educational level was <8 years for 59.9% of subjects and 44.3% were unemployed. The majority of patients had never been married (61.1%) and lived with family (75.8%). Schizophrenic-like disorders represented the main diagnostic category (53.9%), followed by personality disorders (16.1%) and bipolar disorders (12.6%). Among LAIs, FGAs were the first choice in 73.7% of patients. During the six months before initiation, 42.8% of subjects had been hospitalized and 63% had been prescribed oral APs by CMHC psychiatrists. Among this last group, 60% had received APs of the same generation as the LAI, 22% exactly the same active substance.

**Table 2:** Socio-demographic and clinical characteristics of the study cohort.

Characteristics	N (1,070)	*% (100)*
**Sex**		
**Male**	561	*52*.*4*
**Female**	509	*47*.*6*
**Age**		
**18–35**	178	*16*.*6*
**35–64**	727	*68*.*0*
**>64**	165	*15*.*4*
**Employment**		
**Yes**	390	*36*.*5*
**No**	474	*44*.*3*
**Unknown**	206	*19*.*3*
**Nationality**		
**Italian**	986	*92*.*2*
**Non Italian**	84	*7*.*8*
**Marital Status**		
**Never Married**	654	*61*.*1*
**Married**	379	*35*.*4*
**Unknown**	37	*3*.*5*
**Living arrangement**		
**With family**	811	*75*.*8*
**Alone**	191	*17*.*9*
**Residential setting**	47	*4*.*4*
**Other arrangement**	21	*1*.*9*
**Education**		
**< 8 years**	641	*59*.*9*
**> 8 years**	397	*37*.*1*
**Unknown**	32	*3*.*0*
**Living Area**		
**Urban**	591	*55*.*2*
**Non Urban**	479	*44*.*8*
**Diagnosis**		
**Schizophrenic-like disorders**	577	*53*.*9*
**Personality disorders**	172	*16*.*1*
**Bipolar disorders**	135	*12*.*6*
**Depressive disorders**	72	*6*.*7*
**Anxiety disorders**	37	*3*.*5*
**Other disorders**	77	*7*.*2*
**First choice LAI**		
**Fluphenazine Decanoate**	412	*38*.*4*
**Haloperidol Decanoate**	282	*26*.*3*
**Risperidone Long-acting**	234	*21*.*3*
**Zuclopenthixol Decanoate**	81	*7*.*6*
**Paliperidone Palmitate**	45	*4*.*2*
**Perphenazine Enantate**	14	*1*.*3*
**Olanzapine Pamoate**	2	*0*.*2*
**Length of CMHC care before LAI initiation**		
**< = 1 year**	93	*8*.*7*
**> 1 year– 3 years**	204	*19*.*1*
**> 3 years– 10 years**	395	*36*.*9*
**> 10 years**	378	*35*.*3*
**Oral AP in the 6-month period before LAI initiation**		
**Yes**	674	*63*.*0*
**No**	396	*37*.*0*
**Hospital admissions in the 6-month period before LAI initiation**		
**Yes**	458	*42*.*8*
**No**	612	*57*.*2*

### Continuity of LAI treatment and hospital admissions

During the six-month follow-up, 222 patients (20.7% of the cohort) steadily continued LAI treatment, 517 (48.3%) discontinued it definitively, whereas 331 (30.9%) discontinued “intermittently”. Moreover, 646 subjects (60.4%) were prescribed also oral APs during LAI treatment, and 455 (42.5%) received oral AP after the last LAI injection (either as continuation of an oral therapy concomitant to LAIs or as a new treatment). It should also be noted that 97.4% of patients remained in care at CMHC all along the six-month follow-up (only 28 cases, corresponding to 2.6%, were discharged before the end of observation).

As reported in Table 3, use of FGA-LAI (OR_adj_ 1.71; 95%CI 1.18–2.49) and previous hospitalization (OR_adj_ 2.00; 95%CI 1.47–2.74) were associated with LAI continuity All other variables included in the logistic regression model (gender, class of age, nationality, living arrangement, diagnosis) did not result in any statistically significant impact on LAI continuity.

**Table 3:** Predictors of treatment continuity with LAIs over the six-month follow-up.

	OR raw	95%Cl	OR adj	95% CI	P value
M vs F	1.06	0.79–1.43	1.12	0.83–1.52	ns
Age 18–34 vs >64	0.72	0.42–1.25	0.61	0.34–1.10	ns
Age 35–64 vs >64	0.70	0.45–1.07	0.90	0.58–1.38	ns
Immigrants vs Italians	0.82	0.49–1.54	1.02	0.55–1.79	ns
Living alone vs not alone	1.14	0.77–1.65	1.06	0.72–1.57	ns
Schizophrenic-like psychosis vs no psychosis	1.18	0.88–1.60	1.18	0.87–1.59	ns
**FGA-LAI vs SGA-LAI**	**1.58**	**1.11–2.29**	**1.71**	**1.18–2.49**	**< .01**
**Hospital admissions in the six-month period before LAI initiation vs no hospital admissions**	**1.78**	**1.32–2.39**	**2.00**	**1.47–2.74**	**< .001**

During the follow-up, 20% of patients experienced hospitalization for psychiatric reasons compared with 43% in the 6-month period before LAI initiation (p value <0.001). The risk of hospital admission, hence of severe clinical relapse, increased mainly for those patients admitted before LAI initiation (HR 3.20; 95%CI 2.22–4.59, when a time-window of 45 days from the new-start LAI treatment was excluded), for immigrants (HR 3.13; 95%CI 1.28–7.69) and to a less extent in case of free-of-treatment days during the follow-up (HR: 1.14; 95%Cl 1.01–1.97). Also age was found to influence this outcome (p< 0.05), with higher risks for older patients (Table 4).

**Table 4:** Hazard estimation of psychiatric hospitalization over the six-month follow-up: Cox regression model. Different latency time-periods were considered in the columns.

	All days		Latency of 15 days from the new-start LAI treatments		Latency of 30 days from the new-start LAI treatments		Latency of 45 days from the new-start LAI treatments	
	HRadj	95% CI	HRadj	95% CI	HRadj	95% CI	HRadj	95% CI
**No LAI treatment vs LAI-treatment during follow-up (person-days)**	1.17	0.80–1.70	1.29	0.70–1.85	1.40	0.99–1.98	**1.14**	**1.01–1.97**
M vs F	0.93	0.69–1.23	0.85	0.62–1.16	0.89	0.65–1.23	0.89	0.64–1.24
18-34yrs vs >64	0.88	0.61–1.27	0.88	0.60–1.30	0.82	0.55–1.22	0.91	0.60–1.37
**35-64yrs vs >64**	**0.43**	**0.23–0.82**	**0.44**	**0.22–0.89**	**0.42**	**0.21–0.86**	**0.44**	**0.21–0.93**
**Immigrants vs Italians**	**2.50**	**1.22–5.26**	**3.57**	**1.47–9.09**	**3.45**	**1.39–8.33**	**3.13**	**1.28–7.69**
Living alone vs not alone	1.00	0.69–1.45	1.14	0.76–1.72	1.04	0.69–1.57	0.98	0.65–1.48
Schizophrenic-like psychosis vs no psychosis	1.32	0.99–1.76	1.21	0.90–1.65	1.23	0.89–1.68	1.25	0.90–1.72
FGA-LAI vs SGA-LAI	1.06	0.78–1.46	0.86	0.61–1.22	0.85	0.60–1.22	0.80	0.55–1.17
**Hospital admissions in the six-month period before LAI initiation vs. no hospital admissions**	**2.13**	**1.57–2.89**	**2.66**	**1.90–3.71**	**3.00**	**2.11–4.26**	**3.20**	**2.22–4.59**

## Discussion

The annual incidence of treatments with LAIs in Bologna remained substantially unchanged from 2011 to 2015. Only a moderate shift toward initiation with SGA-LAIs was observed, despite other drug-utilization studies documenting the increasing use of these drugs in recent years [8, 11]. It should be mentioned that a large availability of SGA-LAIs was attained in Italy over the study period, but some SGA-LAIs were not yet routinely reimbursed by the Regional Health Service when the study ended (olanzapine, aripiprazole), or until 2014 (paliperidone). It can be argued that pharmaceutical policies of the Regional Health Service have tried to limit the use of these expensive medications, by delaying their inclusion in hospital formularies in order to keep a curb on their direct costs. This could explain the prevalent use of FGA-LAIs in CMHCs of the Bologna area. In 2015 FGA-LAIs (namely fluphenazine and haloperidol) were still chosen in almost two-thirds of new LAI treatments, but paliperidone showed a sharp increase compared to previous years overtaking risperidone, and ended as representing a fifth of new treatments.

Patients with schizophrenic-like disorders were 54% of the study cohort. The remaining proportion was partly represented by bipolar and personality disorders, for which evidence of effectiveness of LAIs is also available [12, 13]. As for cases of depressive and anxiety disorders (10.2% of the cohort), possible off label use should be considered, although also suboptimal diagnostic updating of the database can affect these findings.

In the six-month period before the entry date, less than two-thirds of patients had used some oral APs, at least in terms of out-of-hospital treatments. This rate seems to be low if we consider that guidelines and practices, as well as recent appraisals [14], usually recommend LAIs after stabilization with oral APs. Although it must be acknowledged that the Summaries of Product Characteristics (SPC) of fluphenazine and zuclopenthixol do not explicitly recommend an earlier stabilization with oral AP, previous oral administrations of the same active principle (or of an active principle of the same class) should allow to test the tolerability of medication. An exploration of adverse effects is indeed important, because LAI treatment does not allow to tackle adverse reactions by a rapid reduction in dosage or an immediate discontinuation [9]. Besides, it is well known that clinical effects of LAIs can appear days or weeks after the first administration and overlapping oral APs is recommended, if patient’s compliance allows it. Some specific limitations of data collection should be recognized as partial explanation of these findings. In fact, lack of data on drug prescribed during hospitalizations may affect our analysis contributing to underestimate the number of cases with previous testing for tolerability of APs, which could be in fact occur during a previous hospitalization.

Forty percent of patients of our cohort had been hospitalized with psychiatric diagnosis for at least a day in the six-month period preceding the entry date. This is remarkable, because only 7% of patients treated by Bologna CHMCs have psychiatric admissions in 1-year period. Since hospitalization can be considered a proxy of acute clinical impairment, these data confirm that Bologna CMHCs are more likely to use LAIs for patients with the most severe psychiatric conditions [7].

Although it is widely accepted that LAI administration improves adherence [14–18], in our cohort the proportion of LAI users who discontinued was higher than generally reported [19–21], with only one-fifth of patients remaining on LAI therapy all over the six-month follow-up. This finding could bring again into question [22] whether LAIs actually improve therapeutic adherence or simply allow to know the real patient’s adherence. Since reasons for treatment discontinuation are not recorded, they might be due to poor compliance but also to adverse reactions, as well as to clinical improvements or to increasing therapeutic alliance with CMHC. Our work, in line with a previous study in the same area [7], suggests that CMHC care relies largely on other therapeutic strategies rather than just LAI administrations.

The large proportion of cases (60%) with concomitant oral and long-acting AP medication at follow-up highlighted the well-known tendency of patients with psychiatric diseases to receive AP polypharmacy.

Only two factors were identified through a multivariate analysis as predictors of the six-month continuity of LAI utilization: initiation with FGA-LAI and, most importantly, psychiatric admissions in the previous six-months. As regards the former, this generation is likely to be used in case of higher severity of illness, since some of these agents are deemed to have greater sedative or incisive effect, no matter the scientific evidence and the considerable heterogeneity across single APs. Our findings cast doubts on the alleged lower tolerability of FGA medications, as already highlighted in CATIE and CUtLASS studies [23, 24]. As regards the latter, it seems plausible that patients recently hospitalized have more severe symptoms requiring pharmacological therapy on a longer term. However, it should be noted that surprisingly diagnosis was not associated with LAI continuation, although people with schizophrenic-like disorders are by far the cases for which recommendations of LAI treatment are better defined; therefore, they were expected to exhibit longer-term LAI medication.

A recent hospital admission, being a proxy of clinical seriousness, is also the principal determinant of re-admission at follow-up, although other variables play a part: early discontinuity of LAIs, as admissions after LAI initiation more likely occurred during free-of-treatment days. Nevertheless, our findings show that starting a LAI treatment strongly decreased hospital admissions, while discontinuation had a lower association with next hospitalisation. An expeditious pattern of LAI prescriptions, more crisis-oriented and briefer than expected, may also be evinced by the fact that 555 out of 1,625 cases initiating a new LAI treatment (34.1%) were excluded from the final cohort because they had been in contact with CMHC less than 6 months before initiation.

A few remarks are needed for immigrant subjects: despite their limited number in this study, there was a significant risk of hospitalization at follow-up for this population. Their higher risk of hospital admissions at follow-up could be partially explained by a tendency to provide immigrants with briefer admissions to hospital or residential settings, as showed by a recent 12-month investigation in Bologna, on CMHC patients with severe mental disorders [25]. Combining evidence from these two studies suggests the need of a stricter monitoring of length of admission to health care settings for each subgroup of patients.

The higher risk of hospital admission for psychiatric disorders found for older age could be explained by a general frailty of this population, which may be susceptible to hospitalization also for less severe conditions, in comparison to younger patients.

Both limitations and strengths of this study stem largely from the same source, insofar as data were drawn from the ordinary database of CMHCs and no supplementary clinical information was collected. The short follow-up, the lack of standardized measures of clinical severity, the absence of information on comorbidities, the uncertainty on the actual intake of oral APs supplied by the CMHCs are examples of consequent shortcomings. In addition, an “immeasurable time bias” [26], due to lack of information on drugs prescribed during hospital admissions, may have affected our results on treatment continuity during the follow-up, as well as on exposure to oral APs before LAI treatment and on possible start of LAIs in the hospital. In this regard, a sensitivity analysis was performed assuming LAI administrations during hospital admissions occurred in the follow-up: it confirmed the associations of treatment continuity with use of FGA-LAIs and recent hospitalizations (S1 Table).

On the whole, it may be envisaged that the information retrieved from current databases allows a first, essential level of quantitative and qualitative evidence. Further research with longer follow-up and more detailed information should assess if LAIs can play a transient but effective role in more complex and recovery-oriented strategies of care, favouring and triggering other therapeutic factors, such as a closer involvement of patients and families in person-centred treatments, integrated with talking therapies, psychosocial activities and rehabilitation programs.

In conclusion, the study provides new evidence on LAI utilization in a vast Italian Department of Mental Health. LAIs were used not only for schizophrenic like disorders and not necessarily after previous stabilization with AP therapy. The continuity of treatment seems associated with the severity of clinical symptoms (as it can be inferred by hospital admissions before initiation) and with the choice of FGA-LAIs over SGA-LAIs. The incidence of LAI treatments in CMHCs did not increase along five years, with a surprisingly low proportion of cases with continuous treatment for at least 6 months. Psychiatric hospital admission before LAI initiation was also the main predictor of hospitalization during the follow-up. Studies replicating this analysis in other Departments of Mental Health would allow to compare evidence across different areas and strengthen our findings.

## Supporting information

S1 Table Sensitivity analysis of predictors of treatment continuity with LAIs over the six-month follow-up. LAI administration at hospital admission was assumed.
(PDF)
