# Supplementary material for: Long-acting injectable antipsychotics: Six-month follow-up of new outpatient treatments in Bologna Community Mental Health Centres
Source: PLoS One. 2019 Feb 15;14(2):e0211938. doi: 10.1371/journal.pone.0211938 (PMC6377140; doi:10.1371/journal.pone.0211938)
Supplement: S1 Table — LAI administration at hospital admission was assumed. (PDF) [file pone.0211938.s001.pdf]

S1 Table. Sensitivity analysis of predictors of treatment continuity with LAIs over the six-month follow-up. LAI administration at hospital admission was assumed.

	**OR raw**	**95%Cl**	**OR adj**	**95% CI**	**P value**
M vs F	0.92	0.70-1.22	0.96	0.72-1.28	ns
Age 18-34 vs >64	0.85	0.51-1.42	0.74	0.42-1.30	ns
Age 35-64 vs >64	0.71	0.47-1.05	0.72	0.46-1.09	ns
Immigrants vs Italians	1.23	0.73-2.16	1.13	0.65-2.04	ns
Living alone vs not alone	1.06	0.73-1.51	0.99	0.68-1.42	ns
Schizophrenic-like psychosis vs no psychosis	1.07	0.81-1.41	1.06	0.80-1.42	ns
**FGA-LAI vs SGA-LAI**	**1.64**	**1.17-2.32**	1.84	1.30-2.63	**< .001**
**Hospital admissions in the six-month period before LAI initiation vs no hospital admissions**	**2.02**	**1.53-2.68**	2.28	1.70-3.06	**< .001**
